# Molecular Dioxygen‐Mediated Passivation of Electron Traps in n‐Type Organic Charge‐Transfer Complexes

**DOI:** 10.1002/anie.9080202

**Published:** 2026-06-11

**Authors:** Kirill K. Gubanov, Yana Reva, Christian L. Ritterhoff, Fabio Candolfi, Daniel Langford, Maximilian Herm, Evanie Franz, Ryan W. Crisp, Frank Hampel, Michael Krieger, Andreas Späth, Benjamin Watts, Jörg Libuda, Heiko B. Weber, Bernd Meyer, Dirk M. Guldi, Rainer H. Fink

**Affiliations:** ^1^ Physical Chemistry II Department of Chemistry and Pharmacy Friedrich‐Alexander‐Universität Erlangen‐Nürnberg Erlangen Germany; ^2^ Physical Chemistry I Department of Chemistry and Pharmacy Friedrich‐Alexander‐Universität Erlangen‐Nürnberg Erlangen Germany; ^3^ Computer Chemistry Center (CCC) Friedrich‐Alexander‐Universität Erlangen‐Nürnberg Erlangen Germany; ^4^ Interdisciplinary Center for Molecular Materials (ICMM) Friedrich‐Alexander‐Universität Erlangen‐Nürnberg Erlangen Germany; ^5^ Applied Physics Department of Physics Friedrich‐Alexander‐Universität Erlangen‐Nürnberg Erlangen Germany; ^6^ Interface Research and Catalysis Erlangen Center for Interface Research and Catalysis (ECRC) Friedrich‐Alexander‐Universität Erlangen‐Nürnberg Erlangen Germany; ^7^ Chemistry of Thin Film Materials Department of Chemistry and Pharmacy Friedrich‐Alexander‐Universität Erlangen‐Nürnberg Erlangen Germany; ^8^ Organic Chemistry I Department of Chemistry and Pharmacy Friedrich‐Alexander‐Universität Erlangen‐Nürnberg Erlangen Germany; ^9^ FAU Competence Center Engineering of Advanced Materials (FAU EAM) Friedrich‐Alexander‐Universität Erlangen‐Nürnberg Erlangen Germany; ^10^ Paul Scherrer Institute Villigen Switzerland; ^11^ Center For Nanoanalysis and Electron Microscopy (CENEM) Friedrich‐Alexander‐Universität Erlangen‐Nürnberg Erlangen Germany

**Keywords:** charge‐transport, electron charge‐transfer complex, molecular dioxygen, spectro‐microscopy, trap states

## Abstract

The inherent susceptibility of n‐type organic semiconductors to molecular dioxygen (O_2_) results in electron trapping or in unintended p‐doping, which in turn diminishes their electron mobility. This concept is challenged in the present study by exploring O_2_ interactions with organic charge‐transfer complexes (CTCs), where electron donor–acceptor interactions generate partially delocalized electronic states. Using a CTC comprising a phenazine electron donor and a 7,7,8,8‐tetracyanoquinodimethane (TCNQ) electron acceptor, we demonstrate that its exposure to O_2_ does not lead to electron extraction but instead enhances the charge‐transfer activity. The increased electron density at the TCNQ acceptor upon CTC exposure to O_2_ is attributed to electron trap‐states passivation by O_2_, without evidence of chemisorption. This passivation mitigates recombination losses, resulting in a threefold photoluminescence quantum yield increase, enhanced electrical conductivity, and improved charge‐transfer state efficiency. Similar O_2_‐mediated conductivity enhancements are observed across additional donor–acceptor pairs, proving the broader applicability of this effect, and paving the way for designing O_2_‐enhanced advanced organic electronic materials.

## Graphical abstract

O_2_ enhances charge‐transfer in n‐type organic donor–acceptor charge‐transfer complexes (CTCs) via physisorption‐mediated trap passivation. By suppressing recombination and promoting long‐lived delocalized charge carriers, O_2_ improves electron transport and conductivity without doping. This effect is general across multiple CTCs and highlights a previously unrecognized role of O_2_ in trap passivation of electron‐conducting organic semiconductors.

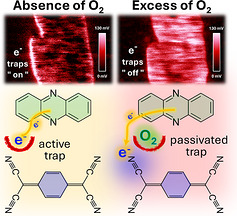

## 1 Introduction

Compared to their p‐type counterparts, n‐type organic semiconductors (OSCs) face significant challenges due to their inherent sensitivity to the ambient conditions [1, 2, 3, 4, 5]. A primary contributor to these limitations is molecular dioxygen (O_2_) [6]. In its triplet ground state, O_2_ possesses a high electron affinity and readily acts as an electron acceptor [7]. In conventional n‐type OSCs, O_2_ extracts mobile electrons and/or localized negative polarons via thermodynamically favorable charge‐transfer interactions, resulting in the formation of superoxide anions ($O_{2}^{-}$) [8]. This process not only reduces conductivity by quenching charge carriers but also initiates spin‐dependent recombination pathways, further intensifying carrier loss [9]. Similarly, O_2_ is known to act as a p‐dopant by accepting electrons from the semiconductor's highest occupied molecular orbital (HOMO), generating positively charged polarons [10]. Although this oxidative doping can be exploited in select cases to enhance hole transport, it generally induces instability and degrades long‐term device performance [11, 12].

While O_2_‐related effects are well‐established in conventional OSCs, the interplay with O_2_ in a distinct class of materials—organic charge‐transfer complexes (CTCs)—remains unexplored [13]. CTCs are formed via intermolecular interactions between electron donors and acceptors, resulting in the emergence of a partial charge‐transfer ground‐state [14, 15]. Unlike single‐component OSCs, CTCs exhibit unique optical and electronic properties originating from intermolecular orbital hybridization and electron density redistribution [16]. These interactions give rise to delocalized electronic states, efficient π‐π stacking, and high charge‐carrier mobility [17]. In CTCs, both n‐ and p‐type behaviors may coexist: majority carriers—electrons—are primarily transported through strongly electron‐withdrawing acceptors, while minority carriers—holes—are localized on the donor units, together enabling efficient charge transport [18, 19].

Though CTCs offer distinct electronic advantages, even highly ordered CTC crystals are subject to structural imperfections formed during the crystal growth [20]. The consequences are both deep and shallow trap states that hinder charge transport and disrupt electron donor–acceptor interactions [21]. While O_2_‐mediated trap‐state effects are well‐studied in conventional OSCs, their role in CTC systems remains poorly understood [22]. Notably, several factors inherent to the charge transport dynamics of CTCs, particularly those with electrons as the dominant carriers, can fundamentally alter the expected interactions with O_2_. On one hand, the lowest unoccupied molecular orbital (LUMO) levels of many electron‐accepting molecules, and thus of the resulting CTCs, frequently reside well below the reduction potential of molecular O_2_, rendering further electron transfer to O_2_ thermodynamically unfavorable [6, 23]. On the other hand, specific centers within electron acceptors (e.g., cyano groups) exhibit exceptionally high electron affinities and, therefore, disfavor any redox interactions with O_2_ [24, 25].

In this study, we document the positive effects of O_2_ on the electronic behavior of a model CTC composed of phenazine (PNZ) as electron donor and 7,7,8,8‐tetracyanoquinodimethane (TCNQ) as electron acceptor [26]. Despite the electron‐rich nature of the complex, we found that O_2_ exposure neither results in electron extraction nor in any redox reaction with CTC. Instead, pronounced enhancements in charge‐transfer activity and electrical conductivity are observed. Supported by a combination of micro‐spectroscopic and electrochemical techniques, we reveal a uniform redistribution of surface potential consistent with reduced electrical resistance upon O_2_ exposure. Spectro‐electrochemical redox probing and density functional theory (DFT) calculations confirmed the absence of chemisorptive O_2_ binding, while electronic structure analyses provide direct evidence of an increased electron density at the cyano‐acceptor orbitals of TCNQ [27]. Remarkably, further analysis showed that the observed conductivity enhancement arises from the physisorptive passivation of electron trap‐states by the O_2_ electron‐rich density [28, 29, 30]. This process suppresses potential charge‐recombination centers, restores effective charge separation, and, hence, reinforces the charge‐transfer character of the complex. To highlight the broader relevance of this phenomenon, we extended our analysis to additional n‐type CTCs, each exhibiting similar O_2_‐induced conductivity improvements. The magnitude of enhancement correlated with electron donor–acceptor strength, suggesting that this effect is common among similar materials. While PNZ and TCNQ are well‐established components in charge‐transfer materials, our findings reveal a beneficial and unexpected role for O_2_ in n‐type CTC systems. These results offer new insights into trap‐states dynamics in electron donor–acceptor materials and pave the way for the rational design of the new O_2_‐compatible and trap‐resilient advanced OSCs [31].

## 2 Results and Discussion

### 2.1 Donor‐to‐Acceptor Ratio in PNZ‐TCNQ‐Based CTCs

**FIGURE 1:**
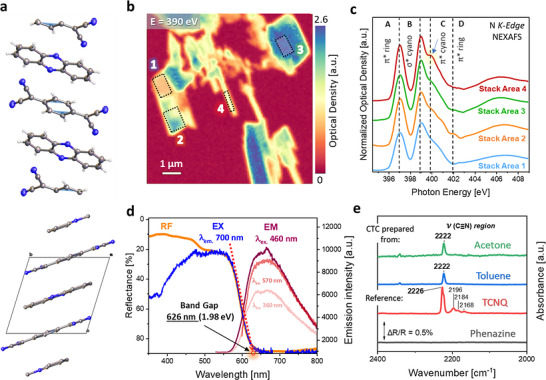
Overview of the investigated CTC system. (a) Molecular packing diagram (top) of the CTC integrated from the single‐crystal XRD, representing the *π*–*π* interactions between the stacks (bottom). (b) STXM micrograph of CTC crystals, recorded at a resonance energy of 390 eV. (c) NEXAFS N *K*‐edge spectra recorded for four different crystal areas, shown in (b). (d) Diffuse reflectance (RF), fluorescence excitation (EX) and emission (EM) spectroscopic characterization of the CTC. (e) ATR‐FTIR spectra of CTC prepared from different solvents in comparison with its precursors.

To understand the structural basis of CTC formation between PNZ and TCNQ, single‐crystal x‐ray diffraction (XRD) analysis was performed (Figure 1a). The resulting structure confirms a well‐ordered 1:1 complex, with alternating PNZ and TCNQ layers stabilized by hydrogen bonding and *π*–*π* stacking interactions [32, 33]. The resolved triclinic unit cell, free of intercalated impurities, correlates with a sandwich‐like packing motif consistent with previously reported models [34]. Visible light microscopy (VLM) reveals distinct morphological differences between the individual components and the CTC crystals (Figure S1). For example, while PNZ forms rod‐like and TCNQ square‐rhombic crystals, the CTC morphology depends on the electron donor/acceptor ratio: equimolar mixtures yield elongated, needle‐like crystals, whereas off‐stoichiometric ratios produce square‐shaped forms. The elongated crystals formed at a 1:1 ratio exhibit the strongest charge‐transfer characteristics [16, 35].

To study the impact of electron donor/acceptor stoichiometry on the CTC electronic structure, we turned to near‐edge x‐ray absorption fine structure (NEXAFS)‐based micro‐spectroscopy. The scanning transmission x‐ray microscopy (STXM) micrograph (recorded in absorption mode) reveals the coexistence of both needle‐like and square‐shaped crystals (Figure 1b), indicative of the heterogeneity in donor–acceptor stoichiometry. Figure 1c shows nitrogen *K*‐edge NEXAFS spectra of the CTC acquired from four regions with different crystal shapes and thicknesses, identified in Figure 1b. Depending on the symmetry of the final state orbitals (an excited electron in an unoccupied molecular orbital), we differentiate between π*‐ and σ*‐resonances. In the present case, four main contributions (the highest intensities compared to the absorption threshold) are distinguished. The compilation of these resonances is given in Table 1 [36]. Two main orbitals are involved in the charge‐transfer: b_3__g_, a_u_ (π* cyano) and, to a weaker extent, b_1g_, b_2u_ (σ* cyano). Notably, the spectrum from the needle‐like crystal (stack area 4) reveals more pronounced resonances corresponding to b_3g_, a_u_ (π* cyano) orbitals (highlighted by an arrow), indicating a higher electron population in these orbitals. This observation suggests a greater degree of electron donation from PNZ to TCNQ in elongated crystals, providing spectroscopic evidence for an enhanced charge‐transfer at equimolar electron donor–acceptor ratios [37].

**TABLE 1:** Compilation of the N *K–*NEXAFS resonances and their assignments retrieved from the TCNQ functional groups according to the building block model.

Peak	Photon energy (eV)	Resonance type	Molecular orbital	Assignation
A	396.8	*π* ^*^	a_u_ b_1u_	Aromatic ring of TCNQ (*π* ^*^ ring)
B	398.7	σ*	b_1g_ b_2u_	In‐plane orbitals of CN‐groups (σ* cyano)
C	399.1	π*	b_3g_ a_u_	Orbitals of CN‐groups (π* cyano)
D	401.8	π*	b_2g_	Highest benzene orbital delocalized over TCNQ (π* ring)

Another noticeable CTC signature is the intense dark red color. It prompts to the absorption of lower energy light due to the partial transfer of electric charges between the electron donor and acceptor. Comparing the diffuse reflectance spectra between CTC, on one hand, and PNZ or TCNQ, on the other hand (Figure S2) assists in understanding the low reflectance and the increased absorbance at 570 nm. This finding confirms that a new electronic state of lower excitation energy is formed. Complementary analysis of the CTC emission unravels 680 nm emission that evolves upon photo‐excitation into the charge‐transfer absorption at 570 nm. Neither PNZ nor TCNQ emits in this spectroscopic range due to their lack of appreciable absorption. Figure 1d summarizes the diffuse reflectance, emission, and excitation spectra of the CTC. An excitation spectrum with 700 nm detection supports the notion that the emission of the charge‐transfer state is excitation‐wavelength‐independent. In fact, excitons relax to the edge of the conduction and valence bands (VBs) via intra‐band cooling, followed by radiative inter‐band recombination [38]. Considering a sizeable overlap in the reflectance and the excitation spectra, that is, in the 520 to 600 nm region, rules out competitive processes other than intra‐band cooling and inter‐band recombination. The difference between the reflectance and excitation spectra between 350 and 475 nm originates from the residual free PNZ and TCNQ. This sets up competing pathways during the light‐absorption, resulting in a lower band‐gap emission intensity. By means of extrapolating the fluorescence excitation spectral edge, we estimate a band gap of 626 nm (1.98 eV) for the CTC. Figure 1e shows the attenuated total reflectance Fourier transform infrared (ATR‐FTIR) spectra of the CTC alongside its precursors, synthesized in both polar and non‐polar solvents. The C≡N stretching vibration is highly sensitive to electronic environment changes and, thus, is an effective probe for monitoring charge‐transfer. Compared to TCNQ, the CTC exhibits a consistent 4 cm^−1^ shift in the C≡N stretching region, regardless of the solvent that was used during the preparation.

### 2.2 Ambient Conditions Impact on CTC Stability and Charge Transport

**FIGURE 2:**
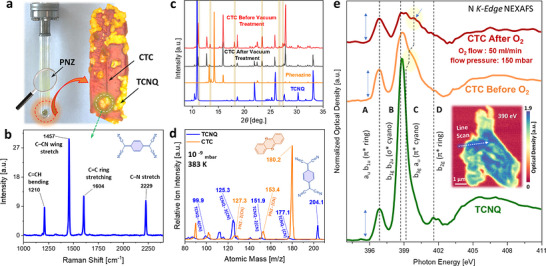
CTC stability examination under high vacuum and O_2_. (a) Illustration of the CTC crystal surface modification in high vacuum with PNZ traces and emerged TCNQ crystals, confirmed by (b) Raman spectroscopy. (c) XRD pattern analysis of the CTC crystal powder before and after vacuum test compared with the precursor materials (yellow markers indicate unique reflections of CTC). (d) QMS spectra of TCNQ and CTC sublimations. (e) In situ NEXAFS N *K*‐Edge spectra of the CTC electronic structure response to O_2_ enriched conditions inside the atmospheric gas‐cell in comparison with TCNQ reference. Spectra are recorded as line scans (indicated with an arrow for CTC crystal in STXM micrograph).

Effective charge‐transfer between two molecular entities depends on the structural stability of the complex, which is affected by a mismatch in vapor pressures between the electron donors and acceptors. It might compromise crystal integrity, especially under high‐vacuum or elevated temperature. TCNQ sublimes at 130°C–150°C and exhibits a relatively low vapor pressure, while PNZ, a heterocyclic compound, sublimes at the lower range of 80°C–100°C [39, 40]. To assess the impact of this difference, we exposed CTC crystal powder to high vacuum (10^−8^ mbar) conditions for more than 120 h. During the evacuation of the CTC, partial redeposition of PNZ was observed on the inner walls of the glass vial, as shown in Figure 2a. Inspection of red CTC crystal surfaces pointed to the emergence of yellow crystalline formations after vacuum exposure. To investigate the composition of the emerged yellow crystals, Raman spectroscopy was performed (Figure 2b). The spectra exhibited characteristic vibrational features of TCNQ. In turn, this confirms that these crystals were mainly composed of the electron acceptor, which is consistent with the fact that PNZ sublimation traces were found on the vial walls [41].

To evaluate the CTC structural integrity after vacuum exposure and subsequent re‐exposure to air, powder XRD analysis was conducted (Figure 2c). While intensity variations were observed, the distinctive diffraction peaks of the CTC (highlighted in yellow) remained intact. Simultaneously, the presence of those diffraction peaks that correspond to TCNQ (10.9°) and PNZ (14.2°) suggests the presence of unbound or segregated components [34].

Insights into the behavior of the individual constituents within the complex under high vacuum came from quadrupole mass spectrometry (QMS) investigation during the sublimation of both pure TCNQ and CTC powders (base pressure: 10^−9^ mbar, sample temperature: 383 K). Figure 2d shows the QMS spectrum of TCNQ with distinct fragmentation peaks associated with the loss of cyano (C≡N) groups. In contrast, the spectrum of the CTC showed a dominant signal at 180 amu, corresponding to the intact PNZ molecule, while the characteristic peak of the intact TCNQ molecule/ion at 204 amu was absent. These findings suggest that PNZ, due to its significantly higher vapor pressure relative to TCNQ, sublimes throughout the early stages of vacuum exposure and dissociates from the complex before TCNQ begins to evaporate. Notably, PNZ sublimation took place at room temperature once the vacuum valve was opened, due to the back pressure in the chamber.

The susceptibility of CTC towards high vacuum led us to explore the effect of the opposite environmental conditions by exposing the CTC crystals to an atmosphere enriched with O_2_. At the forefront was in‐situ NEXAFS‐based micro‐spectroscopic analysis of the CTC electronic structure, conducted within a gas cell into which O_2_ was introduced (Figure 2e). The experimental layout is schematically illustrated in Figure S3 [42]. For the O_2_ exposure experiments, we deliberately selected crystals with a square‐like morphology, which correspond to a lower degree of charge transfer (CT) due to their non‐equimolar electron donor‐to‐acceptor stoichiometries. This choice allowed the changes in the degree of CT to be observed more clearly. From a comparison of the TCNQ and CTC spectra before O_2_ exposure, we conclude that the π* ring resonances (denoted as A) show minimal variation in intensity for both materials. In contrast, the resonances B and C, corresponding to the b_3g_, a_u_ (π* cyano) and b_1g_, b_2u_ (σ* cyano) orbitals, respectively, exhibit significant differences. These orbitals play a central role in the charge‐transfer, and their reduced intensity in CTC relative to TCNQ reflects an increased electron population resulting from the electron transfer.

Upon exposure to O_2_, the intensities of the resonances B and C decrease even further, while the resonance A remains almost unaltered. Notably, both resonances related to the charge‐transfer appear as split spectral features. This effect was observed only when the controlled O_2_ flow reached 50 mL/min at a pressure of 150 mbar. The intensity reduction signifies an increased electron occupation of these previously unoccupied TCNQ orbitals and corroborates an enhancement of the charge‐transfer. The broadening of these orbitals (highlighted by the arrow) implies that additional charge is being accepted by TCNQ [37]. Such a broadening of electronic levels is a clear manifestation of enhanced charge‐transfer activity. In short, the introduction of O_2_ increases the number of electrons accepted by the C≡N orbitals of TCNQ. To rule out potential beam‐induced damage during the NEXAFS measurements, STXM images and a series of NEXAFS spectra were acquired after repeated line scans (Figure S4). The absence of any appreciable changes confirms the structural integrity of the sample throughout the analysis.

While PNZ essentially contributes to the overall electronic structure and charge‐transfer dynamics of the complex, the evaluation of the C≡N orbitals of TCNQ development is sufficient for spectroscopic tracking and understanding of the charge‐transfer characteristics. These molecular orbitals act as primary electron acceptor centers and exhibit distinctive spectral features. Specific information about the electronic transitions and charge redistribution within the TCNQ renders the respective orbitals valuable indicators for monitoring the changes in charge‐transfer activity. In this regard, NEXAFS N *K*‐edge spectral contributions of the PNZ reference material are demonstrated individually (Figure S5).

### 2.3 Nature of O_2_ Interaction With CTC

**FIGURE 3:**
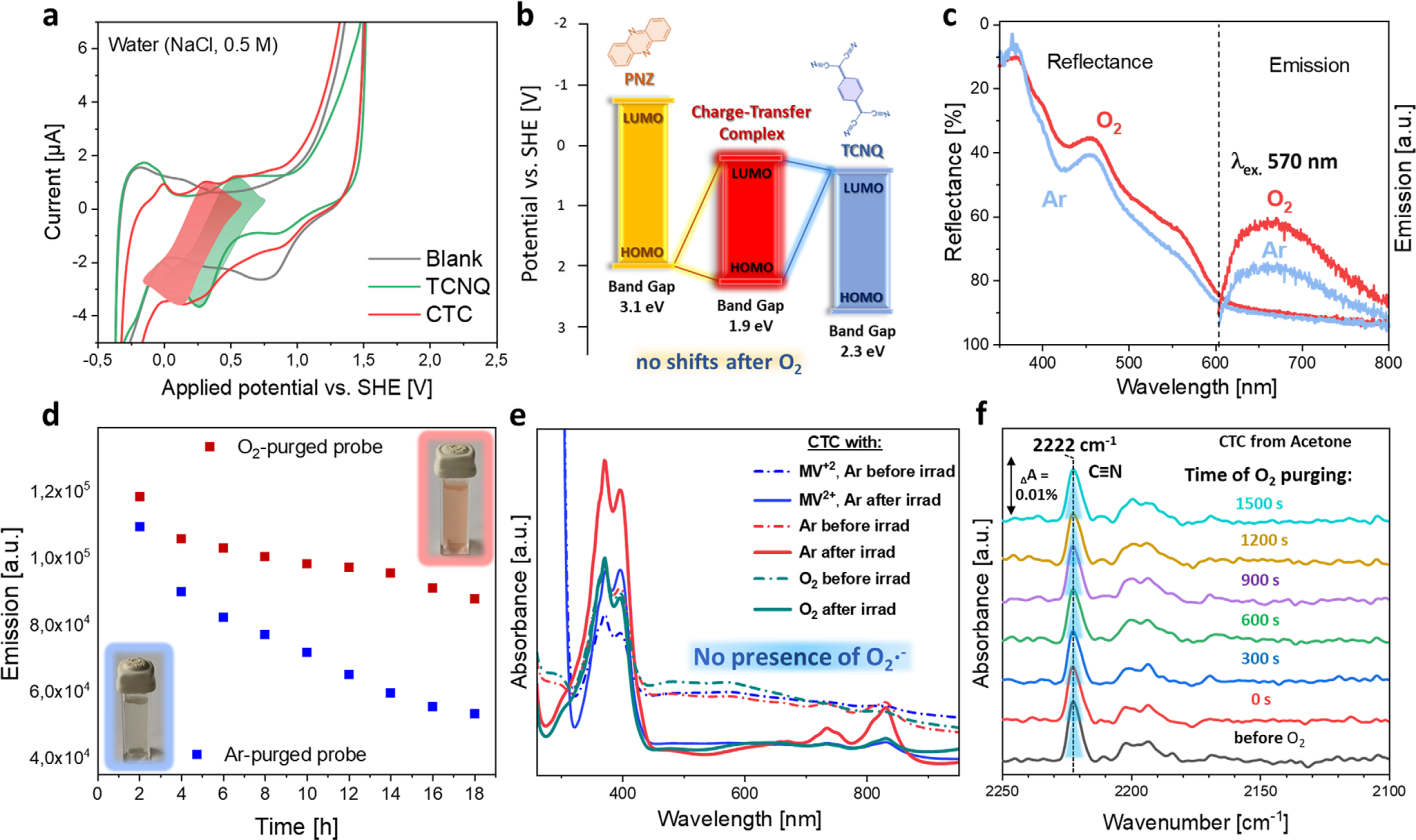
Overview of the O_2_ effect on charge‐transfer in TCNQ‐PNZ‐based CTC. (a) CV in argon‐purged water with 0.5 M NaCl, to determine the CTC conduction band energy. (b) Energy levels of HOMO and LUMO orbitals diagram for CTC and precursor components. (c) CTC emission (excitation 460 nm) and reflectance spectra in the presence of the O_2_ (red) and after purging with argon (blue). (d) The emission stability test for the O_2_ effect on CTC over time. (e) Absorption spectra for CTC dispersion in presence of MV^2+^ reagent. (f) Time‐dependent ATR‐FTIR spectra of CTC during dosing the O_2_.

To investigate the type of interactions between CTC and O_2_, we conducted cyclic voltammetry (CV) (Figure 3a). In aqueous dispersion, the conduction band (CB)/LUMO energy is +0.23 V versus SHE. CV of argon (Ar)‐degassed precursors in acetonitrile solutions (0.1 mM) showed the first TCNQ reduction at +0.38 V versus SHE (Figure S6a) and the first PNZ oxidation at +2.0 V versus SHE, yielding a HOMO–LUMO energy gap of 1.62 eV. This renders CTC formation at 1.98 eV possible. The LUMO of PNZ and HOMO of TCNQ were obtained from reflectance spectroscopy (vide supra) and are put into the energy diagram (Figure 3b). Additionally, electrochemical analysis under O_2_‐rich conditions lacked any shifts in energy levels. Notably, PNZ alone exhibits a reduction at 2.36 V versus SHE (Figure S6b), which shifts in the presence of TCNQ to 1.8 V versus SHE, indicating interaction. Comparing the CTC LUMO of +0.23 V versus SHE with the O_2_ reduction at −0.16 V versus SHE confirms that electron transfer from CTC to O_2_ is thermodynamically unfavorable [43].

To examine the band population, reflectance and emission spectra were recorded for aqueous CTC dispersions under O_2_ and Ar (Figure 3c). After emission intensity correction in accordance with the reflectance, O_2_ exposure led consistently to stronger emission at 570 and 610 nm—indicating enhanced population of the corresponding emissive states. This was noted across the full spectral range, that is, from 350 to 800 nm, in complementary measurements (Figure S7) when using sealed cuvettes that were purged with either O_2_ or Ar for 1 h each. After emission measurements, we observed a gradual fading of the characteristic red color of the CTC dispersions. Complete fading occurred in Ar‐purged samples, while only partial fading was observed in the O_2_‐purged cuvette (Figure 3d). The emission intensity at 610 nm drops in the presence of argon after 14 h and then stabilizes. It reflects the removal of O_2_ from the structural defects. In stark contrast, in the presence of O_2_, changes in the emission intensity are negligible during the initial 14 h and correlate with the CTC stability.

O_2_ is, however, commonly known to act as an electron acceptor in the presence of organic compounds [44]. Therefore, we examined the CTC crystals in the presence of methyl viologen (MV^2+^) upon Ar or O_2_ exposure (Figure 3e). MV^2+^ is known to turn blue with a distinct absorbance at 600 nm in its one‐electron reduced form. Upon 570 nm excitation into the CT absorption of the CTCs, we did not gather any evidence for MV^2+^ reduction, meaning that O_2_ does not undergo any chemisorptive redox reaction (i.e., it does not form O_2_^∙−^ radical anions). A detailed ATR‐FTIR analysis (Figure 3f) further shows no additional bands after O_2_ exposure, confirming the absence of any chemical oxidation. This suggests only weak physisorptive interactions between O_2_ and CTCs.

### 2.4 Electrical Characterization

**FIGURE 4:**
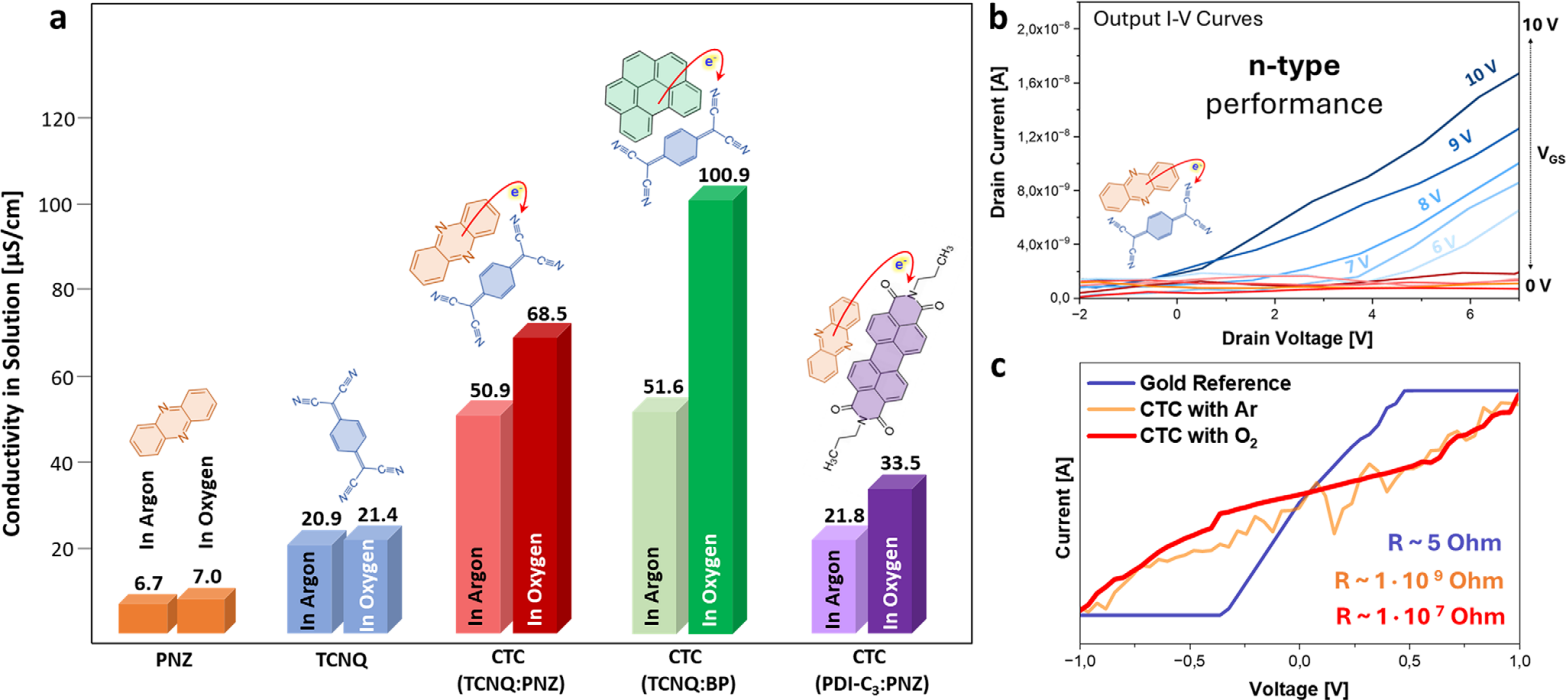
Electrical characterization of n‐type CTC systems. (a) Conductivity variation of different CTCs in solution upon argon and O_2_ exposure. (b) Output I‐V characterization of the TCNQ/PNZ CTCs. (c) Two‐point probe resistance characterization of the TCNQ/PNZ CTC resistances.

The effect of O_2_ on the CTC conductivity was compared with measurements under Ar. To rule out precursor‐specific effects, we also tested benzo(ghi)perylene (BP), a more electron‐rich donor than PNZ, and 2,9‐dipropylanthra[2,1,9‐def:6,5,10‐d'e’f]diisoquinoline‐1,3,8,10(2H,9H)tetrone (PDI‐C_3_), a moderate electron acceptor. Figure 4a shows that O_2_ exposure consistently increases the solution conductivity for all complexes. Measurements were conducted at a fixed temperature of 21.5°C–21.9°C.

Conductivity changes under O_2_ exposure were negligible for the individual precursors (considering the accuracy of conductivity measurements, ±2 µS/cm), but significant for all three CTCs: ∼52% (PNZ/TCNQ), ∼100% (BP/TCNQ), and ∼35% (PNZ/PDI‐C_3_). These differences correlate with the strength of the charge‐transfer interactions, with BP/TCNQ showing the highest degree. For PNZ/TCNQ, the enhanced conductivity remained stable for ∼15 h and was reproducible after 24 h, indicating reversibility and agreement with the emission stability results.

Figure 4b summarizes the electrical characterization of the TCNQ/PNZ CTC crystalline material operating as an n‐type semiconductor in a bottom‐contact organic field‐effect transistor (OFET) configuration. The output I–V characteristics confirm that under a positive gate–source bias and the corresponding direction of charge transport, the TCNQ–PNZ CTCs behave as an n‐type semiconductor, consistent with other TCNQ‐based derivatives [45]. The I–V curves indicate substantial contact resistance and subthreshold leakage current. Both are attributed to a non‐optimized semiconductor‐electrode interface and a high charge‐injection barrier—addressing these device‐level limitations is beyond the scope of this study, given that the central objective is to examine the effect of O_2_ on n‐type CTCs.

In addition to the conductivity increase in solution, we detected a similar effect in the solid crystalline form of the CTCs. To compare the electrical resistance of TCNQ–PNZ CTC crystals in the presence of either Ar or O_2_, two‐point probe measurements with a fixed contact spacing across all samples were done. These measurements were carried out using a Keithley probe station equipped with mechanical micromanipulators, which enabled precise alignment of thin metal probes onto the gold electrodes with the CTC crystals forming a conductive channel. As shown in Figure 4c, the resistance of the CTCs in O_2_ is approximately two orders of magnitude lower than that of the sample measured under Ar. An additional two‐point probe resistance characterization of different electron donor/electron acceptor CTC pairs further proves the broader applicability of the O_2_ effect on CTCs (Figure S8).

**FIGURE 5:**
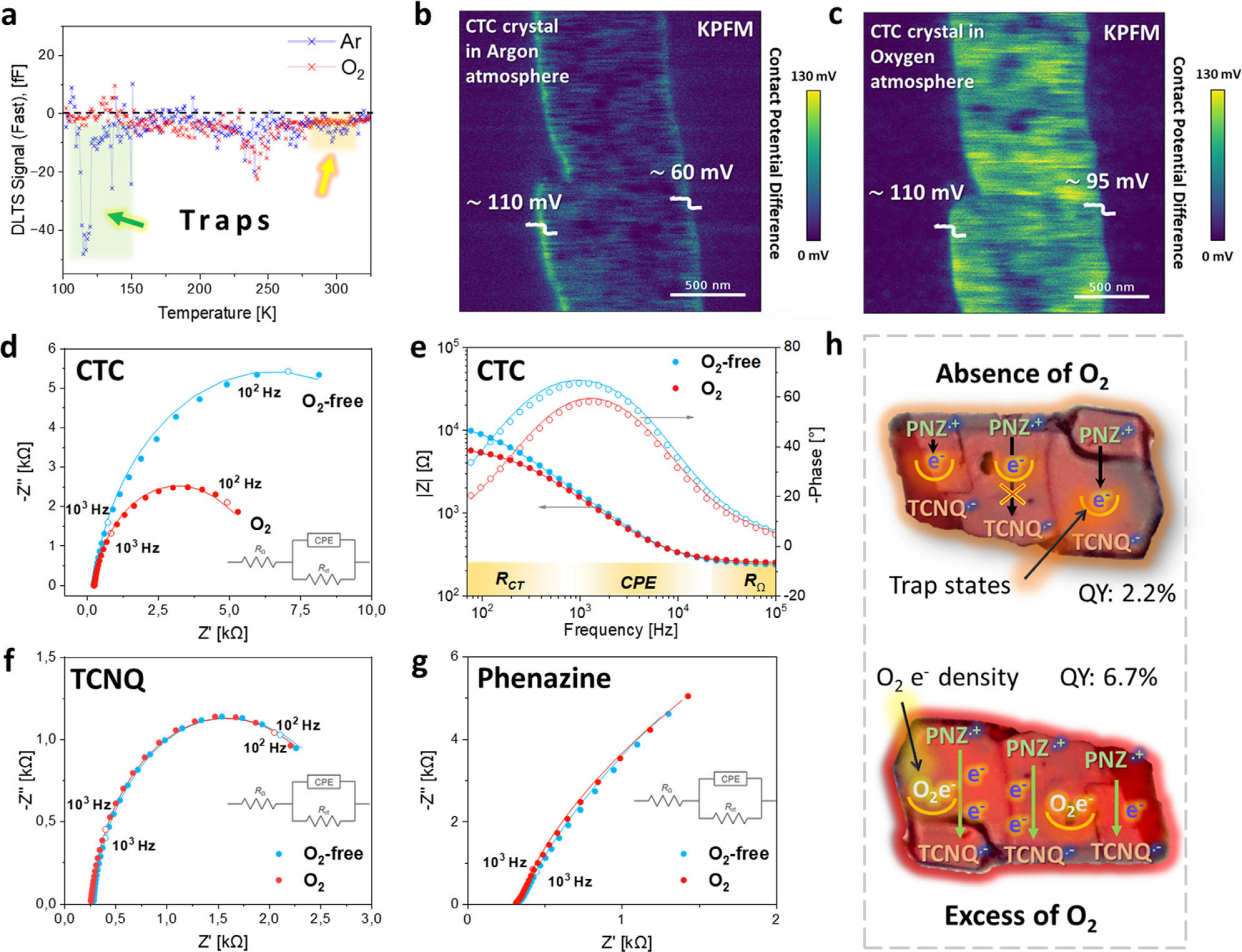
Electrical characterization of CTC. (a) DLTS spectrum taken on a MOS‐like setup with CTC crystals between the SiO_2_ and the gold layer under argon (blue) and O_2_ (red) atmospheres. Negative DLTS signals that are observed in argon atmosphere vanish when measuring the very same sample in O_2_ atmosphere. KPFM CPD maps of the CTC crystal recorded under (b) argon and (c) O_2_. (d) Nyquist and (e) Bode plots of the EIS obtained for 3‐electrode system with Pt‐disc as working electrode, immersed in acetone with 0.2 M TBAPF6 and dispersed CTC; potential was set to +1.3 V (OCP). Nyquist plots of the EIS obtained for 3‐electrode system with Pt‐disc as working electrode, immersed in acetone with 0.2 M TBAPF6 and dispersed (f) TCNQ or (g) PNZ. (h) Mechanism of O_2_ passivating the electron traps, ensuring more efficient charge‐transfer between donor and acceptor pair in CTC.

Investigations with a focus on the presence of potential trap states in the CTCs and their response to molecular O_2_ exposure included capacitive deep level transient spectroscopy (DLTS) measurements [46]. The CTC material was integrated into a metal‐oxide‐semiconductor (MOS)‐like setup, in which gold electrodes were thermally evaporated under vacuum directly onto the CTC crystals (the detailed configuration is provided in the experimental section of supplementary information). DLTS measurements were performed under both Ar and O_2_ atmospheres. A double boxcar correlation function was used to emphasize the initial phase of the transients and is, therefore, sensitive to fast traps (millisecond time scales). The DLTS spectrum (Figure 5a) taken under Ar shows a pronounced negative DLTS signal at around 120 K (green arrow), which completely vanishes in the presence of O_2_. This signal was recorded under a reverse bias of −13 V, subsequent to a pulse voltage of +12 V at the gold top contact with the silicon substrate kept at ground potential. Consequently, electron capture into defect states takes place, resulting in the negative DLTS signal [47]. There is a second weak signal at around 300 K (yellow arrow) corresponding to defects with a larger capture barrier height. In summary, we clearly identified trap‐related signals, which disappear under O_2_ exposure. Note, that the CTC crystals were well accessible to O_2_, in contrast to the more remote and inaccessible Si/SiO_2_ interface.

To uncover the microscopic structural impact on charge transport in the CTC, we turned to contact potential difference (CPD) mapping by means of Kelvin probe force microscopy (KPFM) with simultaneous atomic force microscopy (AFM) imaging. AFM measurements confirm that the morphology of the CTC crystals remains unchanged after exposure to O_2_ (Figure S9). Figure 5b displays the CPD map of the CTC crystal. They show surface potential accumulation and charge localization at the crystal edges prior to O_2_ dosage. According to reported microelectrostatic calculations, strong electron acceptors such as TCNQ feature an effective edge dipole and a large electrostatic potential [48]. These are the likely causes for edge‐localized trap states. Besides, effects of structural reorientations at the crystal edges or lattice defects are not ruled out as a source for charge traps [49, 50]. In contrast, after continuously introducing an excess of O_2_, the charges are redistributed throughout the entire crystal surface (Figure 5c). Control experiments excluded tip‐induced charging (Figure S10) and substrate effects by using a gold‐coated substrate.

Electrochemical impedance spectroscopy (EIS) was applied to a three‐electrode setup immersed in acetone, allowing for the characterization of the electrochemical interface between the Pt‐disc working electrode and the analyte solution [51, 52]. Prior to EIS measurements, the solution was purged with either Ar or O_2_ for 20 min to establish the desired O_2_‐free or O_2_‐saturated environment, respectively. A 0.2 M TBAPF6 electrolyte was added to all analytes to ensure the formation of a stable electrochemical double layer [53, 54]. The applied potential was set to +1.3 V versus SHE, which corresponds to the open‐circuit potential of the CTC‐containing system. This potential ensures thermodynamic equilibrium between the Pt‐disc working electrode and the electrochemical double layer, preventing analyte deposition.

Initially, we conducted measurements in O_2_‐free conditions, comparing the CTC‐containing system with the pristine one (Figure S11). To extract quantitative information, the Randles equivalent circuit model was applied, as it is commonly used to monitor the processes at the interface between the electrode and the electrolyte [55, 56, 57]. Randles circuit consists of several key components: a series resistor (*R*_Ω_) that represents the electrolyte resistance, along with a parallel combination of charge‐transfer resistance (*R*_CT_) and the constant phase element (CPE) to describe a double layer capacitance. Noticeably, *R*_CT_ is significantly reduced in the presence of CTCs and drops from 24.8 to 12.6 kΩ. This implies that the electron‐rich and ‐deficient components within CTC crystals allow to alternate the electrochemical double layer of the working electrode, showing response in the CT region (104 and 102 Hz) of the EIS. Given the demonstrated sensitivity of the electrochemical double layer to the CTC, further tests were conducted to evaluate the impact of O_2_ – Figure 5d,e. According to the Randles model, *R*_CT_ for the O_2_‐free sample reveals resistances of 12.6 kΩ, while it is 5.9 kΩ for the O_2_‐rich sample. This suggests that the presence of O_2_ enhances the CT dynamics by increasing the concentration of charge carriers [58, 59, 60, 61, 62]. In contrast, no such behaviour was observed for the TCNQ reference, where *R*_CT_ remained unchanged regardless of O_2_ (Figures 5f and S12a). Although PNZ shows a decrease in *R*_CT_ from 51.2 to 42.8 kΩ in the presence of O_2_, the insulating nature of the analyte necessitates careful consideration, as its high resistivity impedes the optimization of the fitting (Figures 5g and S12b). Overall, these findings provide strong evidence that O_2_ increases the charge carrier concentration on the electrode surface as a result of its interaction with CTC. All fitting values for the Randles equivalent circuit elements are summarized in Table 2.

**TABLE 2:** Fitting values for the Randles equivalent circuit elements.

	*R*_Ω_/kΩ		*R*_CT_/kΩ		CPE/µΩ^−1^ ∙ s*^n^*	
O_2_ Presence	✓	✗	✓	✗	✓	✗
Blank	—	0.24	—	24.8	—	1.26 _(_ *_n_*_= 0.87)_
CTC	0.25	0.23	5.9	12.6	0.25 _(_ *_n_*_= 0.90)_	0.22 _(_ *_n_*_= 0.90)_
TCNQ	0.26	0.28	2.6	2.6	0.66 _(_ *_n_*_= 0.92)_	0.65 _(_ *_n_*_= 0.92)_
PNZ	0.32	0.33	42.8	51.2	0.34 _(_ *_n_*_= 0.93)_	0.40 _(_ *_n_*_= 0.92)_

Based on experimental evidence, we postulate that O_2_ enhances the charge‐transfer efficiency in the CTCs by passivating intrinsic electron trap states—not chemically, but rather physisorptively—thereby suppressing charge recombination and enabling more effective electron migration toward the TCNQ acceptor sites (Figure 5h). This interpretation is supported by a threefold increase in quantum yield under O_2_ (Figure S13), indicating suppressed non‐radiative losses and improved charge separation.

### 2.5 DFT Calculations

To explore the possible origin of the observed defect states in the CTC compound and their interaction with O_2_, we performed DFT calculations at the hybrid functional level. We considered vacancies and antisite defects on both sublattices as intrinsic defects and H atoms (protons) as a possible source of extrinsic defects.

**FIGURE 6:**
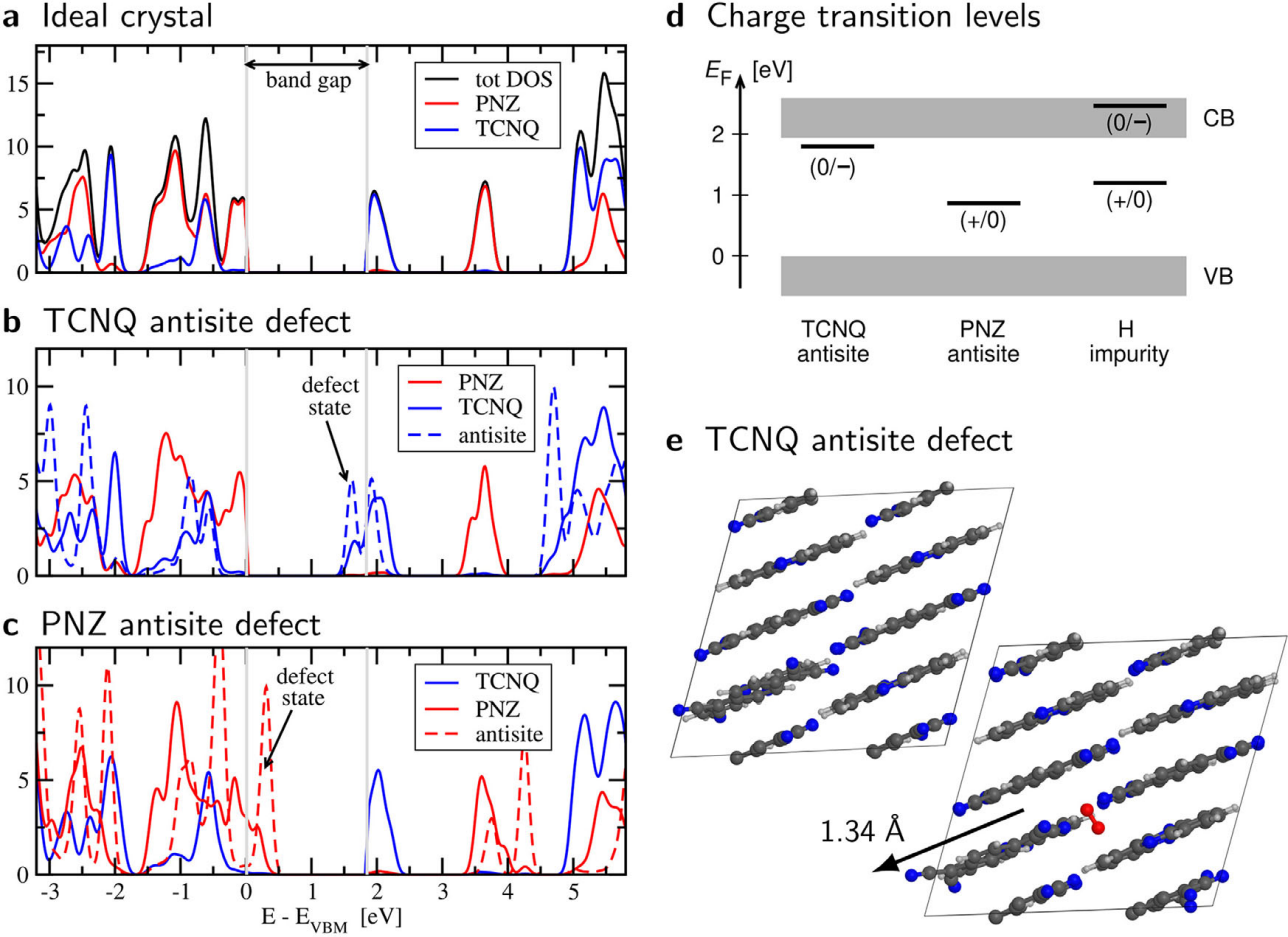
Electronic structure of CTC calculated by DFT at the hybrid functional level of theory. Density of states (DOS) for (a) the ideal CTC crystal, (b) a supercell with a TCNQ antisite defect, and (c) a supercell with a PNZ antisite defect. Solid red and blue lines are the averaged projected DOS of PNZ and TCNQ molecules at regular lattice sites, respectively. Dashed lines are the projected DOS of molecules at the antisite position. The bulk valence band maximum *E*_VBM_ is set to zero. (d) Calculated position of the charge transition levels for different defects relative to the valence band (VB) and conduction band (CB) edges. *E*_F_ is the Fermi energy. (e) Atomic structure of the TCNQ antisite defect before and after insertion of an O_2_ molecule. Solid black lines indicate the size of the supercell.

The density of states for the perfect crystal in Figure 6a shows, as expected for a CTC (Figure 3b), that the VB is formed exclusively by the HOMOs of the electron donating PNZ, whereas the CB states consist only of the LUMOs of the electron accepting TCNQ. In the band structure, we find an indirect band gap of 1.88 eV and an optical band gap at the Γ‐point of 2.26 eV, in good agreement with the experimental band gap of 1.98 eV (Figure 1d). If a vacancy is introduced either on PNZ or on TCNQ sublattice, no significant changes in the electronic structure in the VB and CB region around the band gap are discernible. This changes, however, if antisite defects are considered, that is, when a PNZ vacancy is filled with a TCNQ molecule or vice versa. New electronic states appear in the band gap: acceptor states evolve below the CB for TCNQ antisite defects (Figure 6b) and donor states above the VB for the PNZ antisite defects (Figure 6c). Both defect states are localized at their respective antisite defects with weak delocalization. Novel states in the band gap infer that the antisite PNZs are stronger electron donor and the antisite TCNQs are stronger electron acceptor than the other molecules in the crystal. The charge transition levels in the band gap created by the two antisite defects, as shown in Figure 6d, indicate the Fermi energy positions at which the defect changes its charge state [63, 64]. Hydrogen atoms as extrinsic defects (see Supporting Information) act as both, electron donors and acceptors. In the neutral or negatively charged state, H ‐atoms bind to a TCNQ cyano group and the electrons are delocalized over the whole TCNQ molecule. In contrast, in the positively charged state, protons prefer PNZ sites. Such defects are likely to be the origin of the observed defects with a large capture barrier height in the DLTS measurements (Figure 5a).

For the TCNQ antisite defects, which are more abundant than the PNZ antisite defects according to the stability analysis (Figure 2), we studied the effect of O_2_ incorporation into the lattice. To this end, O_2_ was placed at different interstitial positions followed by structure optimizations. We find that O_2_ neither spontaneously chemisorbs nor reacts with one of the CTC molecules but remains in its triplet state at the interstitial positions. The occupied spin‐up 2π orbitals are well below and the unoccupied spin‐down 2*π* orbitals are well above the band gap (Figure S14). This rationalizes the absence of any electron transfer from O_2_ to the TCNQ antisite acceptors. Moreover, it assists in explaining why neither the electron extraction by O_2_ nor the O_2_^−^ formation is observed in the CTCs, indicating that the TCNQ antisite defects are stronger electron acceptors than O_2_. The preferred O_2_ position is right next to the TCNQ antisite defects where it shifts the latter out of its initial position (Figure 6e). This shift has a strong impact on the position of the defect states in the band gap by shifting them much closer to the CB (Figure S15). Such geometrically induced effects are linked to increased PLQYs (because defect states closer to the band edges reduce Shockley–Read–Hall non‐radiative recombination, which is most significant for mid‐gap states), higher charge‐carrier densities, and increased electrical conductivity after O_2_ treatment.

## 3 Conclusion

In contrast to the well‐established role of molecular dioxygen (O_2_) in conventional n‐type OSCs, where it typically acts as a p‐dopant and/or electron acceptor, our study demonstrates a fundamentally different behavior of O_2_ in organic electron donor–acceptor CTCs governed by electron transfer. Using a model system based on PNZ as electron donor and TCNQ as electron acceptor, we show that O_2_‐rich environments induce regenerative positive effects that enhance charge‐transfer efficiency and electron transport.

NEXAFS‐micro‐spectroscopic evaluation of electronic structure revealed that elongated, needle‐shaped CTC crystals formed at a 1:1 molar ratio between electron donor and acceptor exhibit the highest degree of CT among other crystal conformations. Complementary electrochemical and spectroscopic analyses further identified that O_2_ exposure directly influences the electron‐withdrawing cyano groups of TCNQ involved in the charge‐transfer process, leading to increased population of the VBs and CBs with additional electrons. At the same time, the position of the CTC LUMO and strong electron affinity of the cyano moieties in TCNQ, rule out the reverse process of electron extraction by O_2_, making it thermodynamically unfavorable.

Taking advantage of the solution processability of CTC crystals, we implemented them in a MOS capacitor‐like setup and employed capacitive deep‐level transient spectroscopy to demonstrate that O_2_ exposure effectively passivates both shallow and deep electron trap states. This passivation suppresses potential recombination centers, restores efficient charge separation, and reinforces the charge‐transfer characteristics of the complex. KPFM measurements further confirmed a uniform redistribution of surface potential consistent with reduced electrical resistance, while spectro‐electrochemical redox probing excluded the presence of chemisorptive O_2_ binding.

To assess the generality of this phenomenon, we extended our investigation to two additional n‐type CTCs, all of which exhibited similar O_2_‐induced enhancements in conductivity. The degree of improvement was found to depend on the electron donor–acceptor strength, suggesting a broadly applicable mechanism among comparable materials.

Based on these findings, we propose a mechanism, in which O_2_ passivates electron trap states of CTC, which, under inert conditions, act as potential recombination centers and hinder charge‐transfer efficiency. O_2_ mitigates electron localization by shifting these trap states to the band edges, thereby ensuring the existence of long‐lived, delocalized charge carriers. This process enhances electron migration toward TCNQ acceptor domains, particularly to the electron‐withdrawing cyano groups, leading to a sustained CT and accumulation. Quantum yield measurements support this interpretation, revealing a threefold increase under O_2_ relative to argon, indicative of reduced non‐radiative losses and effective trap‐state mitigation. Although PNZ and TCNQ are well‐established constituents of charge‐transfer materials, our findings reveal a previously unrecognized and beneficial role of O_2_ in modulating electron transport in n‐type CTC systems. These results provide new insights into trap‐state dynamics in electron donor–acceptor materials and open pathways for the rational design of O_2_‐compatible, trap‐resilient advanced OSC systems.

## Author Contributions

**Kirill K. Gubanov**: conceptualization, investigation, validation, writing – review and editing, visualization, writing – original draft, data curation. **Yana Reva**: investigation, formal analysis, writing – review and editing. **Christian L. Ritterhoff**: formal analysis, investigation, validation, writing – review and editing. **Fabio Candolfi**: investigation, formal analysis, writing – review and editing. **Daniel Langford**: investigation, formal analysis, writing – review and editing. **Maximilian Herm**: investigation, formal analysis, writing – review and editing. **Evanie Franz**: investigation, writing – review and editing. **Ryan W. Crisp**: investigation, writing – review and editing. **Frank Hampel**: investigation, validation, formal analysis, visualization, writing – review and editing. **Michael Krieger**: investigation, formal analysis, writing –review and editing. **Andreas Späth**: investigation, writing – review and editing. **Benjamin Watts**: investigation, writing – review and editing. **Jörg Libuda**: validation, writing – review and editing. **Heiko B. Weber**: validation, writing – review and editing. **Bernd Meyer**: formal analysis, validation, writing – review and editing, investigation, visualization. **Dirk M. Guldi**: investigation, writing – review and editing, validation, visualization. **Rainer H. Fink**: conceptualization, funding acquisition, writing – review and editing, project administration, resources, supervision.

## Conflicts of Interest

The authors declare no conflicts of interest.

## Supporting information




**Supporting File 1**: The manuscript version contains available Supporting Information. In particular, all experimental and computational details; VLM of CTC and precursor crystals; compilation of NEXAFS resonances; reflectance spectra of CTC and precursors; layout of gas cell; STXM and NEXAFS spectra of crystal stability; NEXAFS spectrum of PNZ; CV of TCNQ/PNZ and PNZ separate from TCNQ; Emission spectra of CTC; AFM and KPFM of CTC; EIS of CTC and precursors; QY of CTC with Ar and O_2_; DOS plots of other defects.

## Data Availability

The data that support the findings of this study are available on request from the corresponding author. The data are not publicly available due to privacy or ethical restrictions. CIF‐file for x‐ray structural analysis is available online: https://www.ccdc.cam.ac.uk, deposition number: 2481222.

## References

[1] V. Coropceanu , J. Cornil , D. A. da Silva Filho , Y. Olivier , R. Silbey , and J. L. Brédas , “Charge Transport in Organic Semiconductors,” Chemical Reviews 107 (2007): 926–952, 10.1021/cr050140x. 17378615

[2] S. Giannini , A. Carof , M. Ellis , et al., “Quantum Localization and Delocalization of Charge Carriers in Organic Semiconducting Crystals,” Nature Communications 10 (2019): 3843, 10.1038/s41467-019-11775-9. PMC6710274 31451687

[3] A. D. Scaccabarozzi , A. Basu , F. Aniés , et al., “Doping Approaches for Organic Semiconductors,” Chemical Reviews 122 (2022): 4420–4492, 10.1021/acs.chemrev.1c00581. 34793134

[4] I. E. Jacobs and A. J. Moulé , “Controlling Molecular Doping in Organic Semiconductors,” Advanced Materials 29 (2017): 1703063, 10.1002/adma.201703063. 28921668

[5] C. G. Tang , K. Hou , and W. L. Leong , “The Quest for Air Stability in Organic Semiconductors,” Chemical Materials 36 (2024): 28–53, 10.1021/acs.chemmater.3c02093.

[6] P. D. Nayak , B. Dereli , D. Ohayon , et al., “Understanding Oxygen‐Induced Reactions and Their Impact on n‐Type Polymeric Mixed Conductor‐Based Devices,” ACS Central Science 10 (2024): 2229–2241, 10.1021/acscentsci.4c00654. 39735307 PMC11672553

[7] M. Šulka , M. Pitoňák , P. Neogrády , and M. Urban , “Electron Affinity of the O_2_ Molecule: CCSD(T) Calculations Using the Optimized Virtual Orbitals Space Approach,” International Journal of Quantum Chemistry 108 (2008): 2041–2304, 10.1002/qua.21743.

[8] M. Alsufyani , B. Moss , C. E. Tait , et al., “The Effect of Organic Semiconductor Electron Affinity on Preventing Parasitic Oxidation Reactions Limiting Performance of n‐Type Organic Electrochemical Transistors,” Advanced Materials 36 (2024): 2403911, 10.1002/adma.202403911. 39221539

[9] R. Di Pietro , D. Fazzi , T. B. Kehoe , and H. Sirringhaus , “Spectroscopic Investigation of Oxygen‐ and Water‐Induced Electron Trapping and Charge Transport Instabilities in n‐Type Polymer Semiconductors,” Journal of the American Chemical Society 134 (2012): 14877–14889, 10.1021/ja304198e. 22889262

[10] W. Jin , C. Y. Yang , R. Pau , et al., “Photocatalytic Doping of Organic Semiconductors,” Nature 630 (2024): 96–101, 10.1038/s41586-024-07400-5. 38750361 PMC11153156

[11] J. Euvrard , A. Revaux , A. Cantarano , S. Jacob , A. Kahn , and D. Vuillaume , “Impact of Unintentional Oxygen Doping on Organic Photodetectors,” Organic Electronics 54 (2018): 64–71, 10.1016/j.orgel.2017.12.008.

[12] A. Weu , J. A. Kress , F. Paulus , et al., “Oxygen‐Induced Doping as a Degradation Mechanism in Highly Efficient Organic Solar Cells,” ACS Applied Energy and Materials 2 (2019): 1943–1950, 10.1021/acsaem.8b02049.

[13] J. Kirtley and J. Mannhart , “When TTF Met TCNQ,” Nature Materials 7 (2008): 520–521, 10.1038/nmat2211. 18552848

[14] L. Wu , F. Wu , Q. Sun , et al., “A TTF–TCNQ Complex: An Organic Charge‐Transfer System With Extraordinary Electromagnetic Response Behavior,” Journal of Materials Chemistry C 9 (2021): 3316–3323, 10.1039/D0TC05230B.

[15] J. Zhang , W. Xu , P. Sheng , G. Zhao , and D. Zhu , “Organic Donor–Acceptor Complexes as Novel Organic Semiconductors,” Accounts of Chemical Research 50 (2017): 1654–1662, 10.1021/acs.accounts.7b00124. 28608673

[16] K. P. Goetz , D. Vermeulen , M. E. Payne , C. Kloc , L. E. McNeil , and O. D. Jurchescu , “Charge‐Transfer Complexes: New Perspectives on an Old Class of Compounds,” Journal of Materials Chemistry C 2 (2014): 3065–3076, 10.1039/C3TC32062F.

[17] S. Tsuzuki , R. Ono , S. Inoue , S. Matsuoka , and T. Hasegawa , “Origin of the Intermolecular Forces That Produce Donor–Acceptor Stacks in π‐Conjugated Charge‐Transfer Complexes,” Communications Chemistry 7 (2024): 253, 10.1038/s42004-024-01329-6. 39506085 PMC11542100

[18] R. Sanada , D. Yoo , R. Sato , K. Iijima , T. Kawamoto , and T. Mori , “Ambipolar Transistor Properties of Charge‐Transfer Complexes Containing Perylene and Dicyanoquinonediimines,” Journal of Physical Chemistry C 123 (2019): 12088–12095, 10.1021/acs.jpcc.9b01453.

[19] M. Baharfar , A. C. Hillier , and G. Mao , “Charge‐Transfer Complexes: Fundamentals and Advances in Catalysis, Sensing, and Optoelectronic Applications,” Advanced Materials 36 (2024): 2406083, 10.1002/adma.202406083. 39046077

[20] K. P. Goetz , H. F. Iqbal , E. G. Bittle , C. A. Hacker , S. Pookpanratana , and O. D. Jurchescu , “Organic Single Crystals of Charge‐transfer Complexes: Model Systems for the Study of Donor/Acceptor Interactions,” Materials Horizons 9 (2022): 271–280, 10.1039/D1MH01214B. 34679148

[21] H. F. Haneef , A. M. Zeidell , and O. D. Jurchescu , “Charge Carrier Traps in Organic Semiconductors: A Review on the Underlying Physics and Impact on Electronic Devices,” Journal of Materials Chemistry C 8 (2020): 759–787, 10.1039/C9TC05695E.

[22] Y. Huang , K. Wu , Y. Sun , et al., “Unraveling the Crucial Role of Trace Oxygen in Organic Semiconductors,” Nature Communications 15 (2024): 626, 10.1038/s41467-024-44897-w. PMC10799851 38245526

[23] Y. Wang , G. Zhu , E. Zeglio , et al., “Type Organic Electrochemical Transistors With High Transconductance and Stability,” Chemistry of Materials 35 (2023): 405–415, 10.1021/acs.chemmater.2c02447.

[24] M. S. Liu , X. Jiang , S. Liu , P. Herguth , and A. K.‐Y. Jen , “Effect of Cyano Substituents on Electron Affinity and Electron‐Transporting Properties of Conjugated Polymers,” Macromolecules 35 (2002): 3532–3538, 10.1021/ma011790f.

[25] H.‐C. Chen , C.‐P. Hsu , J. N. H. Reek , R. M. Williams , and A. M. Brouwer , “Highly Soluble Benzo[ghi]Perylenetriimide Derivatives: Stable and Air‐Insensitive Electron Acceptors for Artificial Photosynthesis,” ChemSusChem 8 (2015): 3639–3650, 10.1002/cssc.201500950. 26395847 PMC4648035

[26] S. Lee , J. Hong , S. K. Jung , et al., “Charge‐Transfer Complexes for High‐Power Organic Rechargeable Batteries,” Energy Storage Materials 20 (2019): 462–469, 10.1016/j.ensm.2019.05.001.

[27] D. D. T. Mastrogiovanni , J. Mayer , A. S. Wan , et al., “Oxygen Incorporation in Rubrene Single Crystals,” Scientific Reports 4 (2014): 4753, 10.1038/srep04753. 24786311 PMC4007094

[28] Y. Yin , R. Jia , W. Zhang , et al., “Electron‐Rich Oxygen Enhanced Fe‐Doped G‐C_3_N_4_ Mediated Fenton‐Like Process: Accelerate Fe(III) Reduction and Strengthen Catalyst Stability,” Journal of Clean Production 319 (2021): 128680, 10.1016/j.jclepro.2021.128680.

[29] J. K. Pau , M. B. Ruggera , J. K. Kim , and M. C. Caserio , “On the Electron‐Donating Properties of Oxygen vs. sulfur. A Study of the Gas‐Phase Ion Chemistry of Methoxymethylthioalkanes,” Journal of the American Chemical Society 100 (1978): 4242–4248, 10.1021/ja00481a039.

[30] R. J. Thompson , T. Bennett , S. Fearn , et al., “Channels of Oxygen Diffusion in Single Crystal Rubrene Revealed,” Physical Chemistry Chemical Physics 18 (2016): 32302–32307, 10.1039/C6CP05369F. 27849069

[31] K. Ariga , “Materials Nanoarchitectonics at Dynamic Interfaces: Structure Formation and Functional Manipulation,” Materials 17 (2024): 271, 10.3390/ma17010271. 38204123 PMC10780059

[32] J. Wang , J. Miao , C. Jiang , S. Luo , C. Yang , and K. Li , “Engineering Intramolecular π‐Stacking Interactions of Through‐Space Charge‐Transfer TADF Emitters for Highly Efficient OLEDs With Improved Color Purity,” Advanced Optical Materials 10 (2022): 2201071, 10.1002/adom.202201071.

[33] Z. F. Yao , J. Y. Wang , and J. Pei , “Control of π–π Stacking via Crystal Engineering in Organic Conjugated Small Molecule Crystals,” Crystal Growth & Design 18 (2018): 7–15, 10.1021/acs.cgd.7b01385.

[34] I. Goldberg and U. Shmueli , “Structure and Packing Arrangement of Molecular Compounds. III. (1:1) 7,7,8,8‐Tetracyanoquinodimethane–Phenazine,” Acta Crystallographica B 29 (1973): 440–448, 10.1107/S0567740873002724.

[35] C. K. Prout and J. D. Wright , “Observations on the Crystal Structures of Electron Donor‐Acceptor Complexes,” Angewandte Chemie International Edition 7 (1968): 659–667, 10.1002/anie.196806591.

[36] K. Medjanik , D. Chercka , P. Nagel , et al., “Orbital‐Resolved Partial Charge Transfer From the Methoxy Groups of Substituted Pyrenes in Complexes With Tetracyanoquinodimethane—A NEXAFS Study,” Journal of the American Chemical Society 134 (2012): 4694–4699, 10.1021/ja2100802. 22321020

[37] J. Fraxedas , Y. J. Lee , I. Jiménez , et al., “Characterization of the Unoccupied and Partially Occupied States of TTF‐TCNQ by XANES and First‐Principles Calculations,” Physical Review B 68 (2003): 195115, 10.1103/PhysRevB.68.195115.

[38] N. V. Derbenyova , A. A. Konakov , and V. A. Burdov , “Recombination, Multiplication, and Transfer of Electron–Hole Pairs in Silicon Nanocrystals: Effects of Quantum Confinement, Doping, and Surface Chemistry,” Journal of Luminescence 233 (2021): 117904, 10.1016/j.jlumin.2021.117904.

[39] C. G. Kruif and H. A. J. Govers , “Enthalpies of Sublimation and Vapor Pressures of 2,2′‐Bis‐1,3‐Dithiole (TTF), 7,7,8,8‐Tetracyanoquinodimethane (TCNQ), and TTF–TCNQ (1:1),” Journal of Chemical Physics 73 (1980): 553–555, 10.1063/1.439854.

[40] R. D. Chirico , A. F. Kazakov , and W. V. Steele , “Thermodynamic Properties of Three‐Ring Aza‐Aromatics. 1. Experimental Results for Phenazine and Acridine, and Mutual Validation of Experiments and Computational Methods,” J Chem Thermodynamics 42 (2010): 571–580, 10.1016/j.jct.2009.11.010.

[41] M. Yoshikawa , S. Nakashima , and A. Mitsuishi , “Raman Spectra of Thin TCNQ Films Deposited on Silver Films,” Journal of Raman Spectroscopy 17 (1986): 369–371, 10.1002/jrs.1250170414.

[42] T. Huthwelker , V. Zelenay , M. Birrer , et al., “An In Situ Cell to Study Phase Transitions in Individual Aerosol Particles on a Substrate Using Scanning Transmission X‐Ray Microspectroscopy,” Review of Scientific Instruments 81 (2010): 113706, 10.1063/1.3494604. 21133477

[43] J. Li , M. Jiang , H. Zhou , et al., “Vanadium Dioxide Nanocoating Induces Tumor Cell Death through Mitochondrial Electron Transport Chain Interruption,” Global Challenges 3 (2019): 1800058, 10.1002/gch2.201800058. 31565366 PMC6436600

[44] C. Tang , X. Qiu , Z. Cheng , and N. Jiao , “Molecular Oxygen‐mediated Oxygenation Reactions Involving Radicals,” Chemical Society Reviews 50 (2021): 8067–8101, 10.1039/D1CS00242B. 34095935

[45] S. Matsuoka , K. Ogawa , R. Ono , et al., “Highly Stable and Isomorphic Donor–acceptor Stacking in a Family of n‐Type Organic Semiconductors of BTBT–TCNQ Derivatives,” Journal of Materials Chemistry C 10 (2022): 16471–16479, 10.1039/d2tc03634g.

[46] D. V. Lang , “Deep‐Level Transient Spectroscopy: A New Method to Characterize Traps in Semiconductors,” Journal of Applied Physics 45 (1974): 3023–3032, 10.1063/1.1663719.

[47] J. Weber , H. B. Weber , and M. Krieger , “On Deep Level Transient Spectroscopy of Extended Defects in n‐Type 4H‐SiC,” Materials Science Forum 897 (2017): 201–204, 10.4028/www.scientific.net/MSF.897.201.

[48] T. He , Y. Wu , G. D'Avino , et al., “Crystal Step Edges Can Trap Electrons on the Surfaces of n‐Type Organic Semiconductors,” Nature Communications 9 (2018): 2141, 10.1038/s41467-018-04479-z. PMC5976653 29849022

[49] G. D'Avino , L. Muccioli , F. Castet , et al., “Electrostatic Phenomena in Organic Semiconductors: Fundamentals and Implications for Photovoltaics,” Journal of Physics‐Condensed Matter 28 (2016): 433002, 10.1088/0953-8984/28/43/433002. 27603960

[50] H. Jin , E. Debroye , M. Keshavarz , et al., “It's a Trap! On the Nature of Localised States and Charge Trapping in Lead Halide Perovskites,” Materials Horizons 7 (2020): 397–410, 10.1039/c9mh00500e.

[51] D. Kumar , A. Banerjee , S. Patil , and A. K. Shukla , “A 1 V Supercapacitor Device With Nanostructured Graphene Oxide/Polyaniline Composite Materials,” Bulletin of Materials Science 38 (2015): 1507–1517, 10.1007/s12034-015-0966-0.

[52] F. Lisdat and D. Schäfer , “The Use of Electrochemical Impedance Spectroscopy for Biosensing,” Analytical and Bioanalytical Chemistry 391 (2008): 1555–1567, 10.1007/s00216-008-1970-7. 18414837

[53] Q. Huang , Q. Luo , Z. Chen , L. Yao , P. Fu , and Z. Lin , “The Effect of Electrolyte Concentration on Electrochemical Impedance for Evaluating Polysulfone Membranes,” Environmental Science: Water Research and Technol 4 (2018): 1145–1151, 10.1039/C8EW00225H.

[54] A. Nishikata , Y. Ichihara , and T. Tsuru , “Electrochemical Impedance Spectroscopy of Metals Covered With a Thin Electrolyte Layer,” Electrochimica Acta 41 (1996): 1057–1062, 10.1016/0013-4686(95)00438-6.

[55] A. Ch Lazanas and M. I. Prodromidis , “Electrochemical Impedance Spectroscopy─A Tutorial,” ACS Measurement Science Au 3 (2023): 162–193, 10.1021/acsmeasuresciau.2c00070. 37360038 PMC10288619

[56] H. H. Hernández , A. M. R. Reynoso , and J. C. T. González , “Electrochemical Impedance Spectroscopy (EIS): A Review Study of Basic Aspects of the Corrosion Mechanism Applied to Steels,” in Electrochemical Impedance Spectroscopy (IntechOpen, 2020), 10.5772/intechopen.94470.

[57] K. Panigrahi , S. Mal , and S. Bhattacharyya , “Deciphering Interfacial Charge Transfer Mechanisms in Electrochemical Energy Systems through Impedance Spectroscopy,” Journal of Materials Chemistry A 12 (2024): 14334–14353, 10.1039/D4TA00537F.

[58] V. Balasubramani , R. Vignesh , B. Liu , and T. M. Sridhar , “Electrochemical Impedance Spectroscopic Investigation on Detection of H_2_S Gas Using 2D TiO_2_/rGO Nano‐composites,” Applied Surface Science Advances 16 (2023): 100415, 10.1016/j.apsadv.2023.100415.

[59] X. Huang , X. Gao , Q. Xue , et al., “Impact of Oxygen Vacancies on TiO_2_ Charge Carrier Transfer for Photoelectrochemical Water Splitting,” Dalton Transactions 49 (2020): 2184–2189, 10.1039/C9DT04374H. 31998903

[60] J. Zhang , K. Xie , H. Wei , et al., “In Situ Formation of Oxygen Vacancy in Perovskite Sr_0.95_Ti_0.8_Nb_0.1_M_0.1_O_3_ (M = Mn, Cr) Toward Efficient Carbon Dioxide Electrolysis,” Scientific Reports 4 (2014): 7082, 10.1038/srep07082. 25403738 PMC5382710

[61] Y. Liu , L. Kong , X. Guo , J. Xu , S. Shi , and L. Li , “Surface Oxygen Vacancies on WO_3_ Nanoplate Arrays Induced by Ar Plasma Treatment for Efficient Photoelectrochemical Water Oxidation,” Journal of Physics and Chemistry of Solids 149 (2021): 109823, 10.1016/j.jpcs.2020.109823.

[62] J. Bak , H. Bin Bae , and S. Y. Chung , “Atomic‐Scale Perturbation of Oxygen Octahedra via Surface Ion Exchange in Perovskite Nickelates Boosts Water Oxidation,” Nature Communications 10 (2019): 2713, 10.1038/s41467-019-10838-1. PMC6586858 31221958

[63] M. S. Hammer , H. Schlott , L. Lüer , et al., “Bridging Theory and Experiment in Defect‐Tolerant Semiconductors for Photovoltaics,” Nature Reviews Materials 10 (2025): 311–325, 10.1038/s41578-024-00769-9.

[64] C. Freysoldt , B. Grabowski , T. Hickel , et al., “First‐Principles Calculations for Point Defects in Solids,” Review of Modern Physics 86 (2014): 253–305, 10.1103/RevModPhys.86.253.

