# Supplementary material for: Molecular Dioxygen‐Mediated Passivation of Electron Traps in n‐Type Organic Charge‐Transfer Complexes
Source: Angew Chem Int Ed Engl. 2026 Jun 11;65(33):e9080202. doi: 10.1002/anie.9080202 (PMC13452491; doi:10.1002/anie.9080202)
Supplement: Supplementary file 1 [file ANIE-65-e9080202-s001.docx]

**Supplementary Information**

Molecular Di-Oxygen-Mediated Passivation of Electron Traps in n-type Organic Charge-Transfer Complexes

Kirill K. Gubanov ^[a]+^, Yana Reva ^[b]+^, Christian L. Ritterhoff ^[c,j]^, Fabio Candolfi ^[d]^, Daniel Langford ^[b]^, Maximilian Herm ^[b]^, Evanie Franz ^[e]^, Ryan W. Crisp ^[f]^, Frank Hampel ^[g]^, Michael Krieger ^[d]^, Andreas Späth ^[a,h]^, Benjamin Watts ^[i]^, Jörg Libuda ^[e]^, Heiko B. Weber ^[d,j]^, Bernd Meyer ^[c,j]^, Dirk M. Guldi ^[b,j]^ and Rainer H. Fink *^[a,j,k]^

[a]	Physical Chemistry II, Department of Chemistry and Pharmacy, Friedrich-Alexander-Universität Erlangen-Nürnberg, Egerlandstraße 3, 91058 Erlangen, Germany

[b]	Physical Chemistry I, Department of Chemistry and Pharmacy, Friedrich-Alexander-Universität Erlangen-Nürnberg, Egerlandstraße 3, 91058 Erlangen, Germany

[c]	Computer Chemistry Center (CCC), Friedrich-Alexander-Universität Erlangen-Nürnberg, Nägelsbachtraße 25, 91052 Erlangen, Germany

[d]	Applied Physics, Department of Physics, Friedrich-Alexander-Universität Erlangen-Nürnberg, Staudtstaße. 7, 91058, Erlangen, Germany

[e]	Interface Research and Catalysis, Erlangen Center for Interface Research and Catalysis (ECRC), Friedrich-Alexander-Universität Erlangen-Nürnberg, Egerlandstraße 3, 91058 Erlangen, Germany

[f]	Chemistry of Thin Film Materials, Department of Chemistry and Pharmacy, Friedrich-Alexander-Universität Erlangen-Nürnberg, Cauerstraße 3, 91058 Erlangen, Germany

[g]	Organic Chemistry I, Department of Chemistry and Pharmacy, Friedrich-Alexander-Universität Erlangen-Nürnberg, Nikolaus-Fiebiger-Straße 10, 91058 Erlangen, Germany

[h]	FAU Competence Center Engineering of Advanced Materials (FAU EAM), Friedrich-Alexander-Universität Erlangen-Nürnberg, Cauerstraße 3, 91058 Erlangen, Germany

[i]	Paul Scherrer Institute, Forschungsstraße 111, 5232 Villigen, Switzerland

[j]	Interdisciplinary Center for Molecular Materials (ICMM), Friedrich-Alexander-Universität Erlangen-Nürnberg, Nikolaus-Fiebiger-Straße 10, 91058 Erlangen, Germany

[k]	Center for Nanoanalysis and Electron Microscopy (CENEM), Friedrich-Alexander-Universität Erlangen-Nürnberg, Cauerstraße 3, 91058 Erlangen, Germany

*Corresponding Author (e-mail: rainer.fink@fau.de)

^+^Shared First Authors

**Table of Contents**

**Experimental Section.......................................................................3**

**Supplementary Fig. 1……………………………………………………….…………….5**

**Supplementary Fig. 2………………………………………………….………………….5**

**Supplementary Fig. 3…………………………………………………………….……….6**

**Supplementary Fig. 4………………………………………………………….………….6**

**Supplementary Fig. 5…………………………………………………………….……….7**

**Supplementary Fig. 6………………………………………………………….………….7**

**Supplementary Fig. 7…………………………………………………………….……….8**

**Supplementary Fig. 8………………………………………………………….………….9**

**Supplementary Fig. 9………………………………………………………….………….10**

**Supplementary Fig. 10…………………………………………………………….….….10**

**Supplementary Fig. 11…………………………………………………………….……..11**

**Supplementary Fig. 12……………………………………………………………..…….11**

**Supplementary Fig. 13…………………………………………………………………….12**

**Computational Section......................................................................12**

**Supplementary Fig. 14……………………………...…………………………………….13**

**Supplementary Fig. 15……………………………………….…………………………….13**

**References……………………………..……….……………………………………………….14**

**Experimental Section**

**Materials and charge-transfer complex synthesis**

Powders of phenazine (PNZ) and 7,7,8,8-Tetracyanoquinodimethan (TCNQ) were purchased from Sigma Aldrich with 99% purity and used as delivered. The CTC crystals were prepared in acetone (suitable for HPLC, > 99.9% purity) by mixing PNZ and TCNQ in equimolar ratio with concentrations of 30 mM for both compounds. After 2-3 minutes of continuous stirring, the solution color started to change from yellow to red. Two hours later the solution containing crystals was drop cast on filter paper for the subsequent 30 minutes of vacuum filtration. After the crystalline material was collected, single-crystal XRD analysis was performed in order to confirm the donor-acceptor equimolar ratio of the solvent-processed CTC crystals. Di-oxygen was purchased from Westfalen AG (UN 1950 Aerosole). Si wafer substrates with 100 nm thermally grown SiO_x_ were purchased from Siltronic (Munich, Germany). Electrolyte for cyclic voltammetry measurements - Tetra-n-butylammonium hexafluorophos (TBAPF_6_) – was purchased from Sigma-Aldrich with 99.9 % purity.

**Characterization methods and experimental parameters**

Visible light microscopy (VLM) images were recorded with a Leica DM 4000 M microscope equipped with a Leica DFC 320 camera. Atomic Force Microscopy (AFM) and Kelvin Probe Force Microscopy (KPFM) measurements were performed with a JPK Nanowizard 4 in non-contact mode using ElectriMulti75-G cantilever from Budget-Sensors operating at a resonance frequency of 75 kHz and a tip radius below 10 nm. Recorded micrographs have been analyzed with JPK SPM Data Processing software. Quadrupole mass spectrometer (QMS) 400 Balzers was used to monitor evaporation process of the investigated materials and to record their mass spectra. Conductivity in solution was measured using portable conductivity meter COND 7 Vio Set. Cell constant (K*_cell_*) was 1 cm^-1^, with the accuracy of conductivity of ±2 µS/cm and temperature ±0.5 °C. Electrical impedance spectroscopy (EIS) was measured for 3-electrode system with Pt-disc as working electrode, immersed in acetone with 0.2 M TBAPF6 using Gamry Reference 620 potentiostat. Raman spectroscopy was measured with Alpha 300R, Witec microscope at 532 excitation wavelengths, aided with a motorized positioning stage and an ultrasensitive TE cooled spectroscopic camera with backside thinned EMCCD chip. X-Ray Diffraction (XRD) of a single crystal was performed as follows: a suitable crystal with dimensions 0.29 × 0.13 × 0.07 mm^3^ was selected and mounted on a mylar loop in perfluoroether oil on a SuperNova, Dual, Cu at home/near, Atlas diffractometer. The crystal was kept at a constant temperature of 153(2) K during data collection. The structure was solved with the ShelXT n/a (Sheldrick, 2015) solution program using dual methods and by using Olex2 as the graphical interface. The model was refined with ShelXL 2018/3 (Sheldrick, 2015) using full matrix least squares minimisation on F2. The cif file with crystal data is provided additionally. UV-Vis diffuse reflectance spectroscopy was done on a Perkin Elmer Lambda 2 spectrometer or a Perkin Elmer Lambda 35 spectrophotometer. Attenuated Total Reflectance Fourier Transform Infrared Spectroscopy (ATR-FTIR) spectroscopy was done on FT-IR-spectrometer (Bruker Vertex 80v) with gas supply system. θ/2θ XRD patterns were collected on a Bruker D8 Advance with a Cu-Kα source (λ = 1.5418 Å) and a LynxEye XET detector. Near Edge X-ray Absorption Fine Structure (NEXAFS) spectroscopy and Scanning Transmission X-ray Microscopy (STXM) measurements were performed at the PolLux end station of the Swiss Light Source (Paul Scherrer Institute, Villigen, Switzerland) using a carbon-free Au zone-plate with 35 nm outermost zone width. The specimens were prepared onto 100 nm Si_3_N_4_ membranes (Silson Ltd.) by drop-casting. NEXAFS spectra were recorded in so-called “line-scan” mode, which allows for the normalization of the spectra with respect to photon intensities and/or contributions from the specimen support. Spectra and micrographs were analyzed/processed with the aXis2000 software. Potential radiation-induced degradation was minimized and checked carefully. The layout of the atmospheric gas cell is presented in Supplementary Figure S3. The cell is equipped with a temperature and pressure sensors. Gases were prepared by a gas mixing system outside of the chamber and dosed to the cell via Teflon tubes. The O_2_ flow was computer controlled using a regulator as implemented in Labview program for PolLux chamber. Cyclic voltammetry (CV) was carried out in a glass cell with a three-electrode setup: a platinum disc as a working electrode, a platinum wire (5x10 mm) as counter electrode and Ag/AgCl (3 M) as a reference electrode. Applied potential was controlled by a Metrohm μAutolab FraIII and processed by Nova software. All measurements were conducted with scan rate of 100 mV/s. Working electrode was polished after every measurement cycle. Emission and excitation spectroscopy were performed with Shimadzu UV-1900i and Edinburgh FS5 Sectroflurometer, respectively. Fluorescence quantum yields were determined by using an Edinburgh instrument FS5 spectrofluorometer with SC-30 Integrating Sphere Module (measured with ex. 570 nm, indirect in the 2mm cuvette in water dispersion).

For the OFET fabrication, n-doped Si wafer with additional 100 nm thermally grown SiO_2_ on top, served as gate electrode and dielectric, respectively. 40 nm thick gold electrodes were deposited by physical vapor deposition (PVD) through a microstructured mask on the SiO_2_ layer. As final step, a diluted solution of CTC in acetone was drop cast on the electrodes area, so the channel between drain and source electrodes will be filled with recrystallised CTC material, serving as an active semiconductive channel. For the electrical characterisation, the I-V output curves, as well as 2-point probe resistance were recorded using Keithley 4200 parameter analyser.

In capacitive-deep level transient spectroscopy (C-DLTS), the geometry of the setup was the following (from bottom to the top layer): n+-doped Si substrate, 100 nm SiOx, additional octadecyltrichlorosilane (OTS) dielectric layer, TCNQ-PNZ-based CTC, 100 nm of PVD deposited gold electrode. The use of OTS layer served to eliminate potential influence of O_2_ on the silicon oxide surface. The DLTS measurements were performed inside a liquid Air-cooled cryostat in which the atmosphere around the sample could be set. The pressure during the measurement was approx. 5mBar. The DLTS setup is the commercially available FT1030 HERA-DLTS from PhysTech using a Boonton 72B Capacitance Meter. The temperature was monitored using a PT1000 measured by a Keithley 197 DMM. The measurements were performed along increasing temperature starting at the lowest value reachable by the used gas and up to 330K with $\Delta T=1K$. The measurement delay time after the voltage pulse $t_{0}$ was 1.41ms, the time window $T_{W}= 102.91ms$and the pulse time $t_{p}=100\mu s$. The voltage was applied at the metal side and the silicon substrate was grounded. The reverse $V_{R}$ and pulse $V_{P}$ biases were set to $V_{R}=-13V$ and $V_{P}=12V$ respectively. The correlation functions employed are:

$b_{1}=1\cdot\sin\left( \frac{2\pi}{T_{W}}\cdot(t-t_{0}) \right)$	(1)

and the by the DLTS system built-in DLTS-Fast function, which corresponds to a double boxcar correlation function, where the first peak is centered at $\approx t_{0}$ and the second peak at the order of $\approx{2 t}_{0}$, without the function being explicitly specified in the manual.

**Supplementary Figure S1.** VLM micrographs of the (**a**) PNZ and (**b**) TCNQ precursors with (**c**) CTC crystals, prepared from acetone.

**Supplementary Figure S2**. Diffuse reflectance of TCNQ, PNZ and CTC. A new electronic state with a band-edge absorption at 610 nm is formed, which is not present in PNZ or TCNQ. A second band-edge at 490 nm is observable, overlapping with the band-edge of the TCNQ reference, and two small additional maxima at 397 and 375 nm, corresponding with the PNZ reference.

**Supplementary Figure S3**. Atmospheric gas cell equipment layout.

**Supplementary Figure S4**. **NEXAFS-STXM investigation of the x-ray beam damage on the CTC crystal morphology before the O_2_ test.** In order to exclude the beam damage instead of the CTC regeneration effect upon introducing the di-oxygen, STXM micrographs of the CTC crystals were recorded after (**a, d**) the first and (**b, e**) the second line scans. Images after the second line scan were recorded with slightly less amount of points intentionally, in order to minimize the damage, but in overall, the CTC material did not change. Series of the NEXAFS spectra **(c, f)** after the first and the second line scans, showing no spectral changes proving the exclusiveness of the oxygen effect on CTC material upon in-situ measurements.

**Supplementary Figure S5**. **NEXAFS N1s spectra of Phenazine reference material.** The spectral features within 400.1 – 401.3 eV photon energy range have been neglected due to the scanning distortions in this region. Such minor modification however did not affect the overall spectrum representation and following evaluation.

**Supplementary Figure S6. Cyclic voltammetry of precursor components. (a)** CV measurements of TCNQ and PNZ (0.1 mM) solutions (in argon- and O_2_-purged atmosphere). **(c)** CV in argon-purged acetonitrile with 0.5 M LiClO_4_ to compare the band positions of TCNQ and PNZ in separate solutions (each 0.1 mM).

**Supplementary Figure S7**: Emission end excitation spectra show that the emission of O_2_-containing CTC aqueous crystalline dispersion is more intense throughout the whole measurement range (350-800 nm).

**Supplementary Figure S8. Two-point probe resistance characterization of different electron donor / electron acceptor CTC pairs. (a)** Chemical structure of PNZ / PDI-C_3_ CTC. Two-point probe resistance measurements for PNZ / PDI-C_3_ CTC under exposure to **(b)** argon and **(c)** oxygen. **(d)** Chemical structure of TTF / TCNQ CTC. Two-point probe resistance measurements for TTF / TCNQ CTC under exposure to **(e)** argon and **(f)** oxygen. **(g)** Chemical structure of BP / TCNQ CTC. Two-point probe resistance measurements for BP / TCNQ CTC under exposure to **(h)** argon and **(i)** oxygen.

**Supplementary Figure S9**. **Morphology of the investigated CTC crystal for KPFM-oxygen tests.** AFM micrographs of the crystal surface morphology before **(a,b)** exposure to the O_2_ and **(c,d)** after**.**

**Supplementary Figure S10**. **Reverse** **KPFM scan of the CPD map for the CTC crystal before O_2_ dosing.** Scan was performed in 180° reverse direction in order to prove that there is no additional charging from the tip, which stays on the crystal surface.

**Supplementary Figure S11**. **Electrochemical impedance spectroscopy.** **(a)** Nyquist and **(b)** Bode plot of the EIS gathered for 3-electrode system with Pt-disc as working electrode, immersed in acetone with 0.2 M TBAPF6. Potential was set to +1.3V (OCP) and the data was recorded in presence and absence of dispersed CTC. Data are fitted using Randles equivalent circuit model.

**Supplementary Figure S12**. **Electrochemical impedance spectroscopy.** (**a**) Bode plot of the EIS obtained for 3-electrode system with Pt-disc as working electrode, immersed in acetone with 0.2 M TBAPF_6_ and dispersed TCNQ. (**b**) Bode plot of the EIS obtained for 3-electrode system with Pt-disc as working electrode, immersed in acetone with 0.2 M TBAPF_6_ and dispersed PNZ.

**Supplementary Figure S13**. Quantum yield measurements for CTC (**a**) in Ar-purged and (**b**) in O_2_.

**Computational Section**

The DFT calculations were carried out with the plane-wave code PWscf of the Quantum Espresso software package, using Vanderbilt ultrasoft pseudopotentials, and a cutoff energy of 30 Ry for the plane-wave expansion of the wave functions. ^[1,2]^ All structures were relaxed with the gradient-corrected Perdew-Burke-Ernzerhof (PBE) exchange-correlation functional, supplemented by Grimme D3 dispersion corrections. ^[3,4]^ The final electronic structure and the density-of-states (DOS) were determined with the PBE0 hybrid exchange-correlation functional. ^[5]^ All calculations for defect structures with an odd number of electrons (charged defects, hydrogen impurity) and structures including an O_2_ molecule (triplet ground state) were done including spin polarization. For the defect calculations (antisite defects, hydrogen impurity, single O_2_ molecule in the lattice), the bulk unit cell of the PNZ/TCNQ crystal structure was doubled in all three lattice directions, i.e., a (2x2x2) supercell with 8 PNZ and 8 TCNQ molecules (336 atoms) was used. The TCNQ antisite defect was introduced by replacing one PNZ by a TCNQ molecule. Vice versa, the PNZ antisite defect was created by replacing one TCNQ by a PNZ molecule. For the hydrogen impurity and the structures including O_2_ first an extensive screening of their preferred position was performed. A single H atom or a single O_2_ molecule were placed at many different positions in the (2x2x2) supercell and the structure was relaxed. Finally, the electronic structure was analyzed for the lowest-energy configuration. The position of the charge transition levels was calculated from the total energies of the charged and uncharged defects according to the method described in Ref. ^[6]^

**Supplementary Figure S14. Density of states (DOS) for a supercell with an additional H impurity atom.** Solid red and blue lines are the averaged projected DOS of PNZ and TCNQ molecules at regular lattice sites without an additional H atom, respectively. Dashed line is the projected DOS of the TCNQ molecule with the attached H atom. The bulk valence band maximum E_VBM_ is set to zero.

**Supplementary Figure S15. Density of states (DOS) for a supercell with a TCNQ antisite defect after insertion of an O_2_ molecule.** Solid red and blue lines are the averaged projected DOS of PNZ and TCNQ molecules at regular lattice sites, respectively. Dashed line is the projected DOS of the TCNQ molecule at the antisite position. The defect state is shifted to the conduction band edge (compared to **Figure 6b** in the manuscript). The bulk valence band maximum E_VBM_ is set to zero.

**References**

[1] P. Giannozzi, S. Baroni, N. Bonini, M. Calandra, R. Car, C. Cavazzoni, D. Ceresoli, G. L. Chiarotti, M. Cococcioni, I. Dabo, A. D. Corso, S. d. Gironcoli, S. Fabris, G. Fratesi, R. Gebauer, U. Gerstmann, C. Gougoussis, A. Kokalj, M. Lazzeri, L. Martin-Samos, N. Marzari, F. Mauri, R. Mazzarello, S. Paolini, A. Pasquarello, L. Paulatto, C. Sbraccia, S. Scandolo, G. Sclauzero, A. P. Seitsonen, A. Smogunov, P. Umari, R. M. Wentzcovitch, “QUANTUM ESPRESSO: a modular and open-source software project for quantum simulations of materials”, *J. Phys.: Condens. Matter* **2009**, 21, 395502. https://doi.org/10.1088/0953-8984/21/39/395502

[2] D. Vanderbilt, “Soft self-consistent pseudopotentials in a generalized eigenvalue formalism”, *Phys. Rev. B* **1990**, 41, 7892. https://doi.org/10.1103/PhysRevB.41.7892

[3] J. P. Perdew, K. Burke, M. Ernzerhof, “Generalized Gradient Approximation Made Simple”, *Phys. Rev. Lett*. **1996**, 77, 3865. https://doi.org/10.1103/PhysRevLett.77.3865

[4] S. Grimme, J. Antony, S. Ehrlich, H. Krieg, “A consistent and accurate ab initio parametrization of density functional dispersion correction (DFT-D) for the 94 elements H-Pu”, *J. Chem. Phys*. **2010**, 132, 154104. https://doi.org/10.1063/1.3382344

[5] J. P. Perdew, M. Ernzerhof, K. Burke, “Rationale for mixing exact exchange with density functional approximations”, *J. Chem. Phys*. **1996**, 105, 9982-9985. https://doi.org/10.1063/1.472933

[6] M. S. Hammer, H. Schlott, L. Lüer, C. J. Brabec, M. Sytnyk, J. Will, B. Meyer, W. Heiss, “Bridging theory and experiment in defect-tolerant semiconductors for photovoltaics”, *Nat. Rev. Mater*. **2025**, 10, 311-325. https://doi.org/10.1038/s41578-024-00769-9
